# Myeloid-specific blockade of notch signaling alleviates dopaminergic neurodegeneration in Parkinson’s disease by dominantly regulating resident microglia activation through NF-κB signaling

**DOI:** 10.3389/fimmu.2023.1193081

**Published:** 2023-08-23

**Authors:** Shi-Qian Liang, Peng-Hui Li, Yi-Yang Hu, Jun-Long Zhao, Fang-Ze Shao, Fang Kuang, Kai-Xi Ren, Tiao-Xia Wei, Fan Fan, Lei Feng, Hua Han, Hong-Yan Qin

**Affiliations:** ^1^ State Key Laboratory of Cancer Biology, Department of Medical Genetics and Developmental Biology, School of Basic Medicine, Fourth Military Medical University, Xi’an, China; ^2^ Department of Orthopedics, Xijing Hospital, Fourth Military Medical University, Xi’an, China; ^3^ Department of Neurobiology, School of Basic Medicine, Fourth Military Medical University, Xi’an, China; ^4^ Department of Neurology, Tangdu Hospital, Fourth Military Medical University, Xi’an, China; ^5^ Department of Biochemistry and Molecular Biology, School of Basic Medicine, Fourth Military Medical University, Xi’an, China

**Keywords:** notch signaling, Parkinson’s disease, microglia, monocyte-derived macrophages, neuroinflammation

## Abstract

Yolk sac–derived microglia and peripheral monocyte–derived macrophages play a key role during Parkinson’s disease (PD) progression. However, the regulatory mechanism of microglia/macrophage activation and function in PD pathogenesis remains unclear. Recombination signal–binding protein Jκ (RBP-J)–mediated Notch signaling regulates macrophage development and activation. In this study, with an 1-Methyl-4-phenyl-1,2,3,6-tetrahydropyridine (MPTP) hydrochloride-induced acute murine PD model, we found that Notch signaling was activated in amoeboid microglia accompanied by a decrease in tyrosine hydroxylase (TH)–positive neurons. Furthermore, using myeloid-specific RBP-J knockout (RBP-J^cKO^) mice combined with a PD model, our results showed that myeloid-specific disruption of RBP-J alleviated dopaminergic neurodegeneration and improved locomotor activity. Fluorescence-activated cell sorting (FACS) analysis showed that the number of infiltrated inflammatory macrophages and activated major histocompatibility complex (MHC) II^+^ microglia decreased in RBP-J^cKO^ mice compared with control mice. Moreover, to block monocyte recruitment by using chemokine (C-C motif) receptor 2 (CCR2) knockout mice, the effect of RBP-J deficiency on dopaminergic neurodegeneration was not affected, indicating that Notch signaling might regulate neuroinflammation independent of CCR2^+^ monocyte infiltration. Notably, when microglia were depleted with the PLX5622 formulated diet, we found that myeloid-specific RBP-J knockout resulted in more TH^+^ neurons and fewer activated microglia. *Ex vitro* experiments demonstrated that RBP-J deficiency in microglia might reduce inflammatory factor secretion, TH^+^ neuron apoptosis, and p65 nuclear translocation. Collectively, our study first revealed that RBP-J–mediated Notch signaling might participate in PD progression by mainly regulating microglia activation through nuclear factor kappa-B (NF-κB) signaling.

## Introduction

Parkinson’s disease (PD) is a progressive neurodegenerative disease characterized by insidious deterioration of motor control, which often occurs in the older population with emotion, sleep, and cognition disturbances (1). A prominent pathological symptom of PD is the loss of dopaminergic (DA) neurons in the substantia nigra pars compacta (SNpc) and less loss in the ventral tegmental area and other middle brain regions. Although the cause of PD is not fully understood, a large amount of evidence indicates that neuronal degeneration is always accompanied by neuroinflammation (2–5), presented by reactive morphology of microglia and astrocytes, infiltration of monocytes/macrophages, and increased cytokine levels such as tumor necrosis factor-α (TNF-α) and interleukin-6 (IL-6) in cerebrospinal fluid (CSF) and blood (6–10). Therein, innate immune activation, especially microglial activation, represents the major immunologically activated cell population. However, the underlying mechanisms of microglial activation remain unclear.

Microglia, as one kind of classical tissue-resident macrophage in the central nervous system (CNS), originate from the precursors of the embryonic yolk sac and play a pivotal role in cerebral tissue development and neuronal integrity maintenance under physiological conditions (11). However, in several neurogenerative diseases, including PD, microglia are exposed to non-physiological immune activators and become abnormally activated microglia that may promote the pathogenesis and progression of disease (9, 12, 13). Recently, with single-cell RNA sequencing applications, microglia have been shown to be highly heterogeneous, especially during disease progression, and have also been named disease-associated microglia (DAM) (14, 15). DAM is composed of tissue-resident microglia and monocyte-derived macrophages that migrate into the brain *via* the blood−brain barrier. During disease progression, infiltrated monocyte-derived macrophages are intermingled with tissue-resident microglia to speed up or impede disease progression (11). There are at least two subtypes of monocytes in mouse blood: Ly6C^hi^ classical inflammatory monocytes and Ly6C^lo^ non-classical patrolling monocytes, both of which can contribute to infiltrating inflammatory macrophages (IMs) (16, 17). However, how monocyte-derived macrophages and microglia contribute to PD pathogenesis and progression as well as the underlying mechanism are not well defined.

Many studies, including our studies, have shown that recombination signal–binding protein Jκ (RBP-J)–mediated Notch signaling participates in regulating monocyte differentiation and macrophage activation under physiological and pathological conditions (18–20). The Notch signaling pathway is a highly conserved developmental pathway in evolution that regulates cell fate by mediating cell−cell communication. The mammalian Notch signaling pathway consists of four transmembrane receptors, five Notch ligands, the Notch intracellular domain (NICD), and the key transcription factor RBP-J. Once the Notch receptor is activated by its ligand presented by an adjacent cell, the NICD is cleaved by γ-secretase and translocated to the nucleus, where it can associate with RBP-J and then recruit coactivators to trigger downstream gene transcription, such as *Hes1* and *Hes5*, leading to cell proliferation or differentiation (21, 22). Using RBP-J conditional knockout mice combined with some disease models, one of our previous studies suggested that Notch activation in myeloid cells could aggravate spinal cord injury by promoting M1 macrophage polarization and upregulating inflammatory cytokine expression (23). Recently, our study with a mouse experimental autoimmune neuritis model further demonstrated that myeloid-specific Notch signaling activation could alleviate immune-mediated neuropathies by regulating Ly6c^hi^ monocyte conversion through the RBP-J/NR4A1 axis (24). However, how Notch signaling regulates microglial activation and monocyte-derived macrophage infiltration during PD progression remains unknown.

In the present study, we found that the Notch pathway was activated in activated amoeboid microglia in an MPTP-induced PD mouse model. Furthermore, with myeloid-specific RBP-J–deficient (RBP-J^cKO^) mice, we found that myeloid-specific Notch deficiency resulted in more TH^+^ DA neurons and improved movement ability compared with the control PD mice. Meanwhile, the number of resident microglia showed no changes, whereas the number of activated MHC II^+^ microglia and infiltrated monocyte-derived macrophages decreased significantly in RBP-J^cKO^ PD mice. Then, utilizing CCR2^−/−^ mice to block CCR2^+^ monocyte recruitment or a PLX5622-formulated diet to deplete microglia, we found that blockade of CCR2^+^ monocytes contributed negligibly to the attenuated DA neuron degeneration in RBP-J^cKO^ PD mice, whereas microglia depletion enhanced the number of TH^+^ DA neurons and reduced the inflammatory response in RBP-J^cKO^ PD mice. Further mechanistic studies showed that Notch signaling might regulate microglial activation through NF-κB signaling. In summary, our results are the first to reveal that Notch signaling might participate in PD progression by regulating resident microglial activation through NF-κB signaling.

## Method and materials

### Mice and PD models

Wild-type mice with the C57BL/6 background were maintained under specific pathogen–free conditions in the animal facility of the Fourth Military Medical University. For myeloid-specific RBP-J knockout (RBP-J^cKO^) mice, Lyz2-cre (namely, LysM-Cre) transgenic mice (stock #019096, Jackson Laboratory, Bar Harbor, ME, USA) were mated with RBP-J floxed (RBP-J^f/f^) mice (25). After genotype detection, Lyz2-cre^+/−^:RBP-J^+/f^ mice were obtained as the control mice, and Lyz2-cre^+/−^:RBP-J^f/f^ mice were treated as RBP-J^cKO^ mice. The RBP-J knockout efficiency in infiltrated macrophages and microglia was detected with genomic DNA by Real-time Quantitative PCR (qPCR), respectively. CCR2 knockout (CCR2^−/−^) mice (stock #004999, Jackson Laboratory, Bar Harbor, ME, USA) exhibit a defective monocyte recruitment during immune responses and were crossed with RBP-J^cKO^ mice to obtain CCR2^−/−^ RBP-J^cKO^ or CCR2^−/−^ control mice. CX3CR1^GFP^ (stock #005582, Jackson Laboratory, Bar Harbor, ME, USA) mice, which can label CX3CR1^+^ microglia by Green fluorescent protein (GFP) signal, were adopted. In some cases, CX3CR1^GFP^ mice were mated with RBP-J^cKO^ mice. The mouse genotype was determined by polymerase chain reaction (PCR) with mouse genomic DNA. All PCR primers are listed in **Table S1**.

Acute PD models were used in this study. Briefly, MPTP hydrochloride (MPTP-HCl; Sigma Co., St. Louis, MO, USA) was blended in 0.9% sterile saline and then administered to the animals intraperitoneally every 2 h for four times at 20 mg/kg body weight. An equal volume of saline was injected into the control mice. All mouse experiments were approved by the Animal Experiment Administration Committee of Fourth Military Medical University. All animals were treated according to the criteria outlined in the *Guide for the Care and Use of Laboratory Animals* published by the National Institutes of Health.

### Open-field test

Mice were placed in an open-field arena (40 cm × 40 cm × 40 cm) made of white acrylic and monitored using video for 5 min. Four mice in four independent fields were simultaneously recorded. Total distance moved and total time spent in three zones (10 cm × 10 cm, 20 cm × 20 cm for center, and 40 cm × 40 cm for the peripheral zone excluding the center area) were calculated using ETHOVISION 9.0 software (Noldus). After each test, feces were eliminated, and the floor was cleaned with 75% ethanol and then dried completely. The locomotor activity was measured with average speed and moved distance. Exploratory behavior was evaluated as the distance moved in the central area.

### Elevated plus-maze test

The elevated plus-maze apparatus contained four aims (30 cm × 5 cm): two open and two closed arms with the same size, in which 16-cm-high black walls were elevated 45 cm over the floor and weak red light was used as an illuminator. Each mouse was placed in the central square of the plus-maze apparatus and stood facing the open arm, and, then, their behavior was recorded for 5 min. The total number of entries into the open and closed arms, as well as immobility time, was recorded as overall locomotor activity. Meanwhile, the degree of anxiety was calculated according to the percentage of entrance into the open arms.

### Single-cell suspension preparation and FACS

Single-cell suspensions of the brain were prepared according to a previous report (26, 27). Mice were deeply anesthetized in a CO_2_ chamber and transcranially perfused with 20 mL of phosphate buffer saline (PBS). Brains were carefully removed from the skull and ground by Dounce homogenizers. Mononuclear cell isolation was performed by density gradient centrifugation with Percoll (70%/37%). After that, the interphase containing mononuclear cells was collected and washed with 1× HBSS. Myelin was removed by high-speed centrifugation at 850g in a 0.9 M solution of sucrose in 1× Hank's Balanced Salt Solution (HBSS). Mononuclear cells were then rinsed in HBSS. After that, the cells were resuspended completely in PBS containing 0.5% bovine serum albumin (BSA) and 2 mM Ethylenediaminetetraacetic acid (EDTA) and then incubated with antibodies. Each antibody for FACS is listed in **Table S2**.

FACS was performed by BD FACSCanto II. Cell sorting was done using BD FACSAria III. All FACS data were analyzed using FlowJo software (FlowJo LLC).

### Cell culture

The murine microglia cell line N9, the hippocampal cell line HT-22, and the human neuroblastoma cell line SH-SY5Y were cultured in Dulbecco's modified eagle medium (DMEM) (Invitrogen, Carlsbad, CA, USA) containing 2 µM glutamine, 10% fetal bovine serum (FBS), penicillin (100 U/mL), and streptomycin (100 µg/mL). The cells were cultured in a saturated humidified incubator in 95% air and 5% CO_2_ at 37°C. To induce microglial activation, 5 × 10^4^ N9 cells were cultured in 24-well plates and stimulated with Lipopolysaccharide (LPS) (1 µg/mL; *Escherichia coli* 0111: B4, l4391, Sigma-Aldrich, MO, USA) for 24 h.

For primary microglia culture, neonatal mixed culture was slightly modified on the basis of the previous literature (28). postnatal 0–3 (P0–3) mouse pups were anesthetized with hypothermia, the meninges were removed, and the cortices were minced in PBS containing 5% FBS. Cells were then collected by centrifugation and dissociated with trypsin for 20 min at 37°C. After filtration with a 40-µm cell strainer (BD Biosciences, San Diego, CA, USA), the mixed cells were inoculated in 75-mm flasks at a density of 1.5 × 10^7^ and cultured with DMEM containing 10% FBS and Granulocyte-macrophage colony stimulating factor (GM-CSF) (25 ng/mL) (Sigma-Aldrich, St. Louis, MO, USA). After 2 weeks, mixed cells were separated by oscillation (125 Revolutions Per Minute (rpm), 37°C), and, then, microglia were harvested and inoculated in 24-well plates overnight. The next day, the cells were treated with LPS (100 ng/mL) or PBS for 24 h. GM-CSF (25 ng/mL) was added during the whole microglia culture process. The cultured medium and cells were harvested for subsequent enzyme-linked immunosorbent assay (ELISA) detection and RNA preparation. For the detection of p65 translocation, microglia were inoculated on a slide in a 24-well plate and treated with LPS (100 ng/mL) for 6 h followed by immunofluorescence staining.

For BMDM culture, bone marrow cells were isolated from mouse femurs and were cultured in DMEM containing 10% FBS for 16h, and then the suspension cells were inoculated in 24-well plate at a density of 2 × 106 and cultured with DMEM containing 10% FBS and GM-CSF (25 ng/mL) for 7 days to obtain BMDMs.

### Coculture experiments

LPS-treated primary microglia with neuron were cocultured. Primary microglial cells (3 × 10^5^) were cultured in 24-well plates and then stimulated with LPS (100 ng/mL) for 6 h. After washing with fresh DMEM, the activated microglia were cocultured with 5 × 10^4^ HT-22 cells for 36 h, and, then, the mixed microglia and HT-22 cells were collected and stained with CD45 and Annexin V/Propidium Iodide (PI) (Sigma-Aldrich, St. Louis, MO, USA) for FACS analysis.

Coculture primary microglia with N-Methyl-4-Phenylpyridinium Iodide (MPP)(+) induced DA neurons. MPP(+) neuron/microglia coculture experiment was slightly modified on the basis of the previous literature (29). In brief, SH-SY5Y (8 × 10^4^) cells were cocultured with primary microglia cells (3 × 10^5^) in 24-well plates. MPP^+^ iodide (Selleck, Houston, TX, USA) (1 μM) was applied directly to the mixed cultures for 48 h, and, then, the mixed microglia and SH-SY5Y cells were collected and stained with CD45 and Annexin V/PI (Invitrogen, Carlsbad, CA, USA) for FACS analysis.

### Immunofluorescence

Mice were sacrificed and transcranially perfused with 30 mL of PBS plus 30 mL of 4% paraformaldehyde (PFA). Brains were fixed again in 4% PFA for 4 h followed by 30% sucrose dehydration overnight. Frozen sections were made using a cryostat microtome (Leica, Nussloch, Germany). A series of coronal sections (14 µm) containing the midbrain were cut and attached to gelatine-coated slides. After drying at room temperature, sections were blocked with blocking buffer (1% bovine serum albumin plus 0.3% Triton X-100 in PBS) for 2 h at room temperature. Primary antibodies were incubated with sections at 4°C overnight. The next day, secondary antibodies were incubated with the sections for 1 h at 37°C. Hoechst 33258 was counterstained (Sigma-Aldrich, St. Louis, MO, USA) for 15 min. Therein, the DA neurons in the SNpc were stained using anti–tyrosine hydroxylase (TH) (1:10,000) (Sigma-Aldrich, St. Louis, MO, USA) antibody, and microglia in the SNpc were stained using anti–ionized calcium-binding adaptor molecule-1 (IBA-1) (1:1,000) (Wako, Kyoto, Japan) antibody or anti-Transmembrane Protein 119 (TMEM119) (1:200) (Abcam, Cambridge, UK) antibody. All sections were observed and photographed using a fluorescence microscope (BX51, Olympus, Tokyo, Japan) or a laser scanning confocal microscope (FV1000, Olympus, Tokyo, Japan). TMEM119 antibody information is listed in **Table S2**.

### Quantification of TH^+^ neurons and CX3CR1^+^/NICD microglia

We identified the SN regions according to the mouse atlas of Franklin and Paxinos (30) and quantified the SN regions corresponding to −3.64 to −2.92 on the bregma axis. The total number of TH^+^ neurons of the SN was determined on the basis of the stereological methods that are described in published literature (31). In brief, a total of 10 sections were taken at intervals of 5 after consecutive sections, and, then, TH immunohistochemical staining was performed. The total number of TH^+^ neurons in the SNpc from the 10 tissue sections was counted to quantitatively analyze the whole number of DA neurons in the midbrain of the right hemisphere. For microglia counting, the section at bregma of −3.08 was selected, in which there was the most prominent microglia activation. All CX3CR1^+^ cells in the photographed field were counted, and, then, the mean value was analyzed.

### Real-time PCR

Total RNA was extracted according to the protocol using TRIzol reagent (Invitrogen, Carlsbad, CA, USA). Quantitative real-time PCR was performed using an SYBR Premix EX TaqTM II kit (Takara Bio, Dalian, China) and the ABI PRISM 7500 real-time PCR system, and β-actin was used as an internal control. The primers used for qPCR are listed in **Table S1**.

### Enzyme-linked immunosorbent assay

The concentrations of TNF-α, IL-1β, IL-6, IL-10, and transforming growth factor-β (TGF-β) in mouse serum and cell culture supernatant were determined with ELISA kits (eBioscience, San Diego, CA, USA) according to the recommended procedures. Each sample was measured in triplicate.

### Western blot

Cells were lysed in Radioimmunoprecipitation assay (RIPA) buffer buffer containing the protease inhibitor Phenylmethanesulfonyl fluoride (PMSF) (Beyotime, Shanghai, China), and, then, nucleic and cytoplasmic protein extraction kits were applied (Beyotime, Shanghai, China). Protein concentrations were quantitated with a Bicinchoninic Acid Assay (BCA) Protein Assay kit (Pierce, Waltham, MA, USA). Samples were run by sodium dodecyl sulfate polyacrylamide gel electrophoresis (SDS-PAGE) and the membrane was blocked with 5% skim milk for 1 h and then incubated with the primary antibodies and secondary antibodies. Protein was determined with the Ultra High Sensitivity ECL Kit. All antibodies are listed in **Table S2**.

### PLX5622 administration

Mice were fed PLX5622 formulated in the AIN-76A diet for microglia depletion (1,200 parts per million (ppm); Plexxikon) according to a previous description (32, 33). A standard AIN-76A diet was provided as a control diet [standard diet (SD)]. Mice were fed PLX5622 for 7 days to deplete microglia and fed SD as a control. After that, the mice were treated with MPTP and fed the PLX5622 diet or Standard Deviation (SD) for 7 consecutive days.

### Statistics

Data were analyzed with GraphPad Prism version 9 (San Diego, CA, USA). Image-Pro Plus 6.0 software (Media Cybernetics Inc., Bethesda, MD, USA) was used for quantification analysis. The statistical analyses were performed with Student’s t-test or one-way ANOVA with Tukey’s multiple comparisons test. The results are shown as the mean ± Standard Deviation. P < 0.05 was statistically significant.

## Results

**Figure 1:**
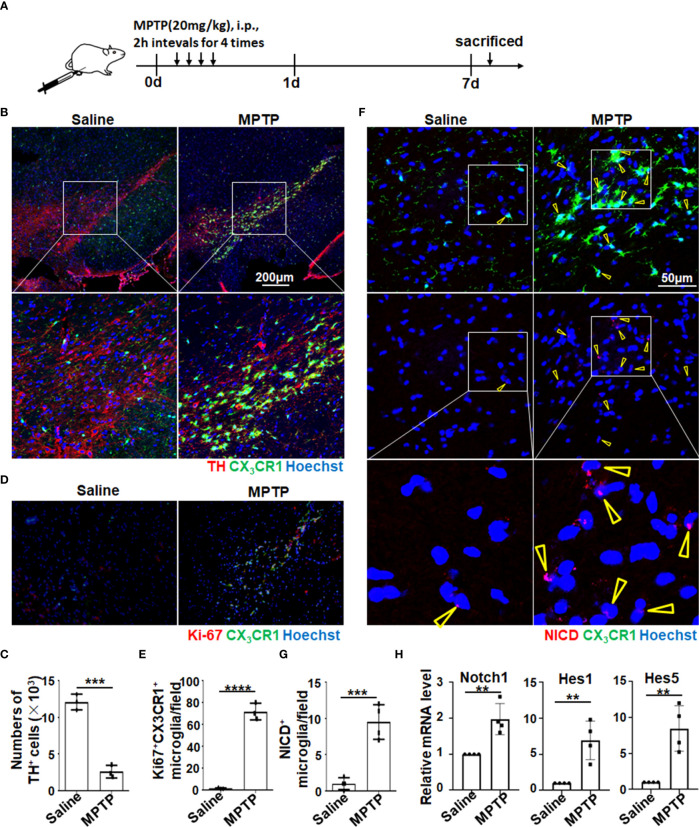
Notch signaling was activated in amoeboid microglia in MPTP-induced PD mice. **(A)** Mice were treated intraperitoneally with MPTP-HCl (20 mg/kg) or PBS every 2 h for four times and then sacrificed on day 7 for subsequent analysis. **(B)** Representative immunofluorescence images of tyrosine hydroxylase (TH) staining in the SNpc of CX3CR1^GFP/+^ mice suffering from PD and control mice. **(C)** The TH^+^ neurons in **(B)** were quantitatively compared (n = 3). **(D)** Representative immunofluorescence images of Ki-67 staining in the SN of CX3CR1^GFP/+^ PD mice and control mice. **(E)** The Ki67^+^ CX3CR1^+^ microglia in **(D)** were measured using Image-Pro Plus and then quantitatively compared (n = 3). **(F)** Representative immunofluorescence images of Notch intracellular domain (NICD) expression in the SN of CX3CR1^GFP/+^ PD mice and control mice. **(G)** The NICD-activated microglia in **(F)** were counted and quantitatively compared (n = 4). **(H)** Mononuclear cells of the whole brain were isolated by gradient centrifugation using 70%/30% Percoll. The mRNA expression of Notch-related molecules (Notch1, Hes1, and Hes5) was determined by qRT-PCR (n = 4). The Student’s t-test was used for the statistical analyses. Bars = mean ± SD; **P < 0.01; ***P < 0.005 ****p< 0.0001..

Notch signaling could be activated in microglia of PD mice. Many studies, including ours, have reported that Notch signaling can regulate macrophage activation and function (34–38). To assess whether Notch signaling can be activated in microglia in PD, we first established an acute PD model with MPTP treatment using CX_3_CR-1^GFP/+^ mice, in which CX3CR1^+^ microglia can be traced by GFP signal (**Figure 1A**). Then, immunofluorescence staining of brain sections was performed to observe the pathological phenotype of MPTP mice. As shown in **Figures 1B, C**, TH^+^ DA neurons were reduced significantly in the SNpc, where microglia (CX3CR1^+^ or TMEM119^+^) adjacent to TH^+^ neurons transformed to amoeboid activated microglia as previously reported (11) (**Figures 1B, D**, **Figure S1A**). Meanwhile, FACS analysis further identified that CD11b^+^CD45^lo^ microglia were indeed tissue-resident microglia by staining with CX3CR1, TMEM119, F4/80, and Ly6C (**Figures S1B, C**). Furthermore, more proliferated CX3CR1^+^ microglia in PD mice were confirmed by Ki-67 staining (**Figures 1D, E**). Enthusiastically, compared with the control mice, more NICD translocated into the nuclei of microglia in PD mice accompanied by the reduced TH^+^ DA neurons, suggesting that Notch signaling was activated in microglia of PD mice (**Figures 1F, G**, **Figure S1E**). This result was further supported by a higher expression of Notch signal–related molecules, such as Notch1, Hes1, and Hes5, in the brains of PD mice (**Figure 1H**). Collectively, these results indicated that Notch signaling was activated in microglia of MPTP-induced acute PD mice.

### Myeloid-specific RBP-J deficiency alleviated dopaminergic neurodegeneration in MPTP mice

**Figure 2:**
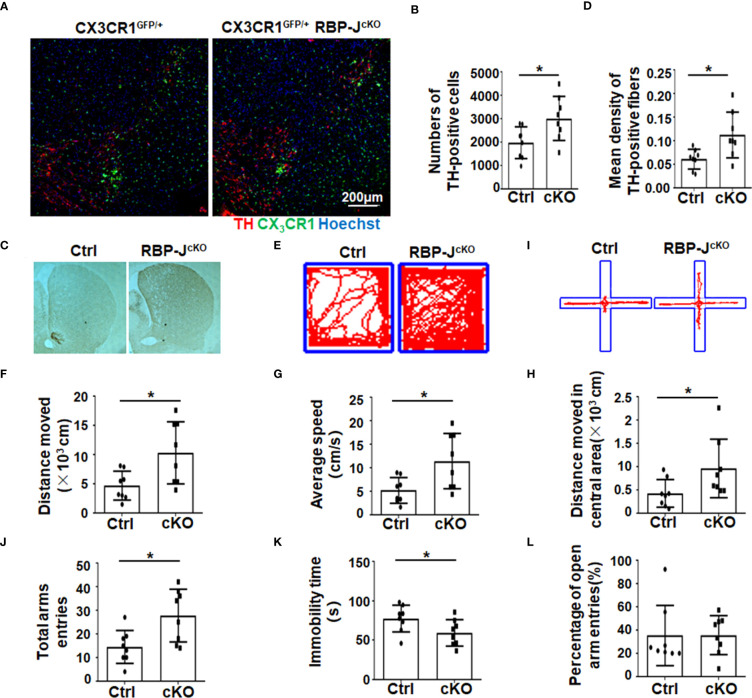
The disruption of RBP-J in myeloid cells alleviated dopaminergic neurodegeneration after MPTP treatment in mice. **(A)** Representative immunofluorescence images of TH staining in the SN of CX3CR1^GFP/+^RBP-J^cKO^ and CX3CR1^GFP/+^ (Ctrl) mice after MPTP treatment. **(B)** The number of TH^+^ neurons was counted and quantitatively compared (n = 8). **(C)** Representative immunohistochemistry staining of TH in the striatum of RBP-J^cKO^ and control PD mice (n = 8). **(D)** The density of TH^+^ axon fibers in striatum was measured by IOD/area with Image-Pro Plus and quantitatively compared (n = 8). **(E-H)** The open-field experiment was performed **(E)**. Locomotor activity—namely, distance moved **(F)**, average speed **(G)**, and distance moved in the central area—was counted and quantitatively analyzed between RBP-J^cKO^ and control (Ctrl) PD mice (n = 8). **(I-L)** The elevated plus-maze test was performed **(I)**. The total arm entries **(J)**, immobility time **(K)**, and percentage of open arm entries **(L)** were calculated and quantitatively compared. The Student’s t-test was used for the statistical analyses. Bars = mean ± SD; *P < 0.05.

Next, to address whether activated Notch signaling in microglia/macrophages could influence PD progression, Lyz2-cre^+/−^:RBP-J^f/f^ (RBP-J^cKO^) mice, in which Notch signaling was specifically blocked in myeloid cells, were adopted. In some cases, RBP-J^cKO^ mice were crossed with CX3CR1^GFP^ mice. After MPTP treatment, the number of TH+ DA neurons in the SNpc was recorded by immunofluorescence staining in CX3CR1^GFP/+^RBP-J^cKO^ and CX3CR1^GFP/+^ PD mice. The results showed that the number of TH-positive cells in myeloid-specific RBP-J–deficient mice was greater than that in control mice (**Figures 2A, B**). Correspondingly, the mean density of TH-positive axon fibers in the striatum of RBP-J^cKO^ PD mice was higher (**Figures 2C, D**). Moreover, the movement behaviors of the mice were examined using the open-field test and the elevated plus-maze test. In the open-field test, RBP-J^cKO^ mice showed better movement ability, as reflected in the moved distance, average speed, and the moved distance in the central area (**Figures 2E-H**). In the elevated plus-maze test, RBP-J^cKO^ mice also presented better movement behaviors based on the total time of entries and immobility count, but there was no obvious difference in the percentage of open arm entries compared with that of the control PD mice (**Figures 2I-L**). Together, these results demonstrated that the disruption of RBP-J in myeloid cells could alleviate DA neurodegeneration in PD mice.

### Myeloid-specific RBP-J deficiency inhibited microglial activation and reduced the inflammatory response in PD mice

**Figure 3:**
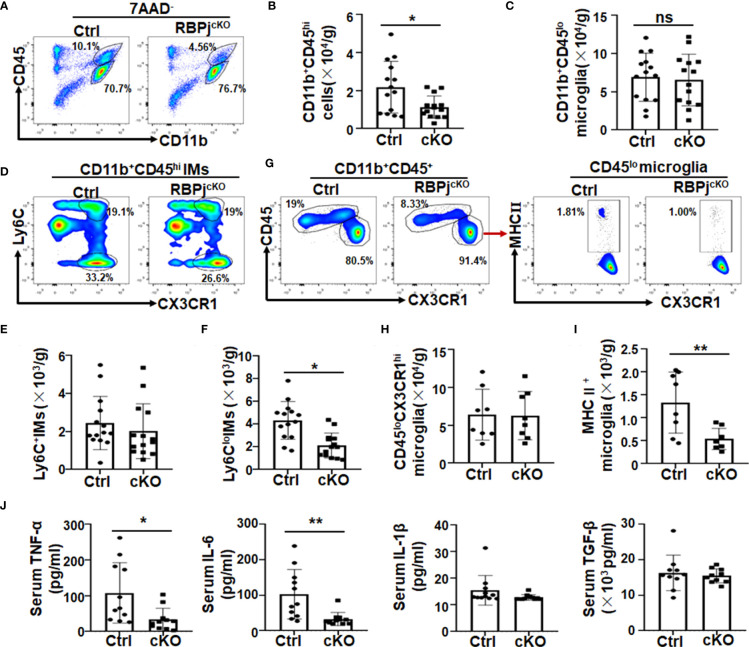
Myeloid-specific RBP-J deficiency inhibited microglial activation and reduced the inflammatory response in PD mice. Single-cell suspensions were prepared from the brains of RBP-J^cKO^ and control (Ctrl) PD mice. **(A)** The myeloid cell population were analyzed by FACS. **(B, C)** The number of CD11b^+^CD45^hi^-infiltrated inflammatory macrophages (IMs; B) and CD11b^+^CD45^lo^ microglia **(C)** in **(A)** were analyzed and quantitatively compared, respectively (n = 14). **(D)** Infiltrated IMs were further analyzed with Ly6c and CX3CR1 staining by FACS. **(E, F)** The total cell number of CD11b^+^CD45^hi^CX3CR1^+^Ly6c^+^ **(E)** and CD11b^+^CD45^hi^CX3CR1^+^Ly6c^lo^ **(F)** IMs in brain was quantitatively compared (n = 14). **(G)** The microglia (CD11b^+^CD45^lo^CX3CR1^hi^) and the activated microglia (MHCII^+^CD11b^+^CD45^lo^CX3CR1^hi^) were analyzed by FACS. **(H, I)** The total cell number of microglia **(H)** and activated microglia **(I)** in **(G)** was quantitatively compared; **(J)** The levels of IL-6, TNF-α, IL-1β, and TGF-β in serum of RBP-J^cKO^ and Ctrl PD mice were detected using ELISA (n = 11 in IL-6, TNF-α, and IL-1β; n = 10 in TGF-β). The Student’s t-test was used for the statistical analyses. Bars = mean ± SD; *P < 0.05; **P < 0.01 ns, no significance.

Because of the role of inflammation in neurodegeneration (3, 11, 12, 39), we next analyzed the phenotype of immune cells, especially myeloid cells, in RBP-J^cKO^ and control PD mice by FACS (**Figure 3A**, **Figure S2A**). Meanwhile, as shown in **Figure S3**, the RBP-J knockout efficiency in sorted CD11b^+^CD45^hi^-infiltrated IMs could reach more than 50%, whereas that in sorted CD11b^+^CD45^lo^ microglia was around 25%. Consequently, the FACS results indicated that the number of CD11b^+^CD45^hi^ IMs was significantly reduced in myeloid-specific RBP-J–deficient PD mice compared with control PD mice (**Figures 3A, B**). The total cell number of CD11b^+^CD45^lo^ microglia showed no difference between the two groups (**Figure 3C**, **Figure S2**), although the microglia numbers increased both in RBP-J^cKO^ and control mice following MPTP treatment (**Figures S2B, C**). Expectedly, Notch signal blockade in myeloid cells showed no effect on microglial proliferation and apoptosis, as demonstrated by Ki-67 and Annexin V staining (**Figure S3**). Because IMs in the brain have been reported to originate from blood monocytes, we further confirmed their phenotype with more cell surface markers, such as Ly6C and CX3CR1 (16, 26, 40–42). The FACS analysis showed that the number of Ly6C^+^CX3CR1^+^ IMs showed no difference between the two groups, but the Ly6C^lo^CX3CR1^+^ IMs decreased obviously in myeloid-specific RBP-J–deficient mice (**Figures 3D–F**), suggesting that RBP-J deficiency in myeloid cells could mainly affect the differentiation of Ly6C^lo^ IMs during PD progression. Because more activated microglia occurred during PD progression (**Figure 1D**) and RBP-J deficiency in myeloid cells did not influence the total cell number of microglia, we proposed that Notch signaling might regulate microglial activation. As expected, the MHC II^+^ activated microglia in RBP-J^cKO^ PD mice indeed showed a marked decrease (**Figures 3G–I**). Consistently, proinflammatory cytokines, including TNF-α and IL-6, in the serum of RBP-J^cKO^ PD mice showed a remarkable decrease, whereas anti-inflammatory cytokines such as TGF-β showed no difference between the two groups (**Figure 3J**), and IL-10 was undetectable (data not shown). Collectively, these results indicated that myeloid-specific RBP-J deficiency alleviated neuroinflammation and DA neurodegeneration, which might be attributed to the decreased number of infiltrated IMs and less activated microglia during PD progression.

### CCR2 depletion contributed less to the attenuated dopaminergic neurodegeneration in myeloid-specific RBP-J–deficient mice

**Figure 4:**
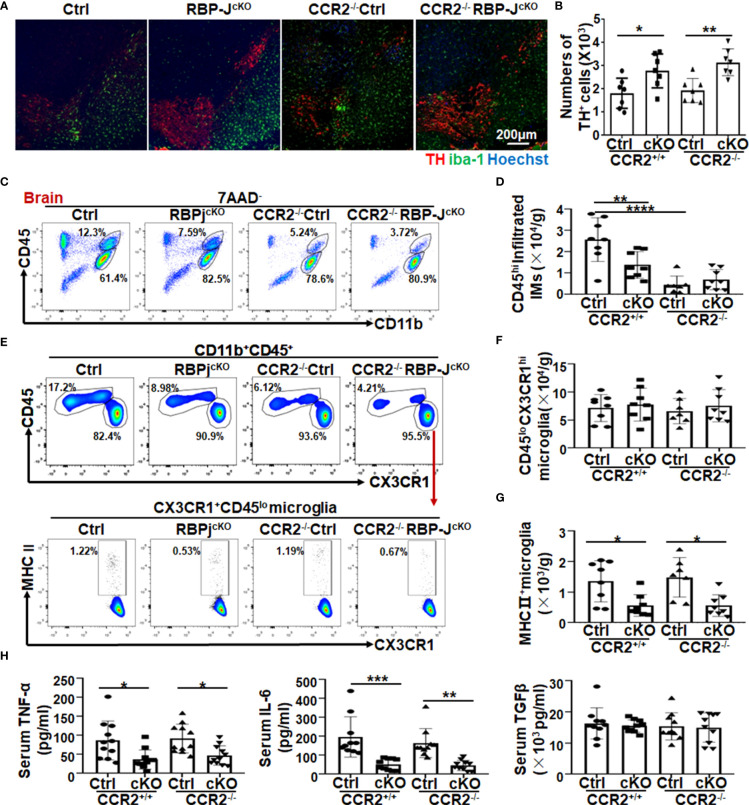
CCR2 depletion contributed less to the attenuated dopaminergic neurodegeneration in myeloid-specific RBP-J–deficient PD mice. **(A)** Ctrl, RBP-J^cKO^, CCR2^−/−^, and RBP-J^cKO^/CCR2^−/−^ mice were treated intraperitoneally with MPTP-HCl as mentioned above to induce the acute PD model. Brains were dissected and stained with TH, IBA-1, and Hoechst on tissue sections using immunofluorescence staining. **(B)** The numbers of TH^+^ neurons were counted in five areas and quantitatively compared among each group (n = 8). **(C)** Single-cell suspensions were prepared from the brain, and infiltrated IMs were analyzed by FACS. **(D)** The CD11b^+^CD45^hi^-infiltrated IMs in **(C)** were quantitatively compared (n = 7 in the CCR2^−/−^group; n = 8 in the other groups). **(E)** The total microglia and activated microglia were analyzed by FACS. **(F, G)** The total cell number of microglia **(F)** and MHCII^+^ activated microglia **(G)** in **(E)** was quantitatively compared (n = 7 in the CCR2^−/−^group; n = 8 in the other groups). **(H)** The levels of TNF-α, IL-6, and TGF-β in serum among each group were measured using ELISA (n = 11 in TNF-α; n = 10 in IL-6 and TGF-β). One-way ANOVA with Tukey’s multiple comparisons test was used for the statistical analyses. Bars = mean ± SD; *P < 0.05; **P < 0.01; ***P < 0.001; ****P < 0.0001.

Ly6C^hi^ monocytes are recruited to the CNS in a CCR2-dependent manner (43–45). Combined with our previous studies, in which myeloid-specific Notch signaling blockade ameliorated murine renal fibrosis and lung fibrosis by regulating CCR2^+^ monocyte-derived macrophage recruitment (37, 38), we wondered whether the attenuated DA neurodegeneration in RBP-J^cKO^ mice also depended on the reduction in CCR2^+^ monocyte recruitment. To address this question, we crossed RBP-J^cKO^ mice with CCR2^−/−^ mice to gain RBP-J and CCR2 double-knockout mice (CCR2^−/−^RBP-J^cKO^), in which the migration of CCR2^+^ monocytes was blocked. After MPTP treatment, FACS assays showed that the Ly6C^hi^ monocytes in peripheral blood were dominantly diminished in CCR2^−/−^ and CCR2^−/−^RBP-J^cKO^ mice when compared with the Ly6C^hi^ monocytes in control and RBP-J^cKO^ mice, whereas Ly6C^int-lo^ monocytes increased in CCR2^−/−^ and CCR2^−/−^RBP-J^cKO^ mice (**Figures S4A, B**), indicating that CCR2 deficiency can successfully deplete blood Ly6c^hi^ monocytes but not Ly6C^int-lo^monocytes. Next, immunofluorescence staining showed that CCR2 deficiency could not enhance the numbers of TH^+^ DA neurons in the SNpc between RBP-J^cKO^ and CCR2^−/−^RBP-J^cKO^ mice (**Figures 4A, B**). Meanwhile, compared with the control PD mice, the numbers of TH^+^ DA neurons were not changed in CCR2^−/−^PD mice, which was consistent with previous reports in which CCR2 blockade does not prevent striatal dopamine loss in the MPTP-induced PD model (46–48). Moreover, although CD11b^+^CD45^hi^-infiltrated IMs decreased significantly in CCR2^−/−^ PD mice compared with control mice, CCR2 knockout did not result in significantly decreased IMs in RBP-J^cKO^ PD mice (**Figures 4C, D**), indicating that myeloid-specific RBP-J deficiency alleviated DA neurodegeneration independent of CCR2^+^ monocyte recruitment. More importantly, regardless of whether CCR2 was knocked out, the total number of MHC II^+^ activated microglia was decreased significantly in the RBP-J^cKO^ mice (**Figures 4E–G**). Meanwhile, the expression of some inflammatory factors, including TNF-α and IL-6, was also decreased significantly in the serum of RBP-J^cKO^ mice or CCR2^−/−^RBP-J^cKO^ mice. However, the level of the anti-inflammatory factor TGFβ showed no difference among the groups (**Figure 4H**). Together, these results indicated that CCR2^+^ monocytes might not contribute to the attenuated DA neurodegeneration in RBP-J^cKO^ PD mice.

### Microglia depletion increased TH^+^ neuron cells slightly in myeloid RBP-J deficient PD mice

**Figure 5:**
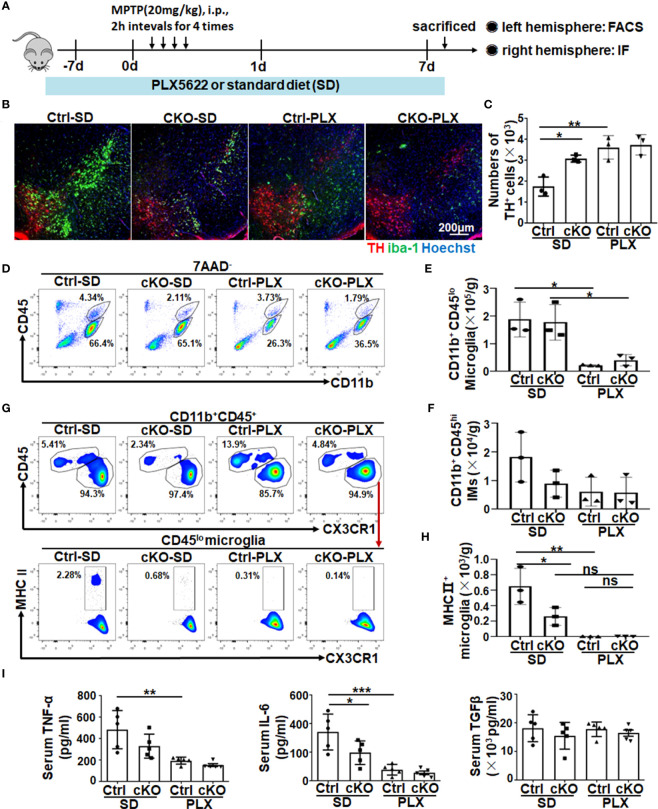
The depletion of microglia showed a resistance role in neurodegeneration of myeloid-deficient RBP-J PD mice. **(A)** Mice were fed a commercial PLX5622 diet or standard diet for 7 days and then treated with MPTP to induce a PD model accompanied by a PLX5622 diet for another 7 days. **(B)** Brain sections were made from RBP-J^cKO^ or control mice fed the above diet and then subjected to immunofluorescence staining with anti-TH and IBA-1 antibodies. Nuclei were stained with Hoechst (n = 3). **(C)** The number of TH^+^ neurons in **(B)** was counted and quantitatively compared among each group (n = 3). **(D)** Single-cell suspensions from the brains of PD mice in **(A)** were prepared and analyzed by FACS (n = 3). **(E, F)** The number of microglia **(E)** and infiltrated myeloid cells **(F)** in **(D)** was quantitatively compared among each group (n = 3). **(G)** MHC II^+^ microglia from different groups were analyzed by FACS (n = 3). **(H)** The number of MHC II^+^ microglia was quantitatively compared among each group (n = 3). **(I)** The levels of TNF-α, IL-6, and TGF-β in serum among each group were detected using ELISA (n = 5). One-way ANOVA with Tukey’s multiple comparisons test was used for the statistical analyses. Bars = mean ± SD; *P < 0.05; **P < 0.01; ***p< 0.001; ns, no significance.

As mentioned above, Notch signaling might regulate microglial activation during PD progression, and we further depleted microglia by feeding mice commercial food containing a small-molecule inhibitor of CSF1R signaling, namely, PLX5622, to assess its contribution (32, 33, 49, 50). First, we confirmed the efficiency of microglia depletion by feeding mice a PLX5622-formulated AIN-76A diet (PLX) or AIN-76A diet [standard diet (SD)] for 7 days. The results showed, *via* immunofluorescence staining and FACS assays, that PLX5622 administration effectively depleted Iba1^+^ microglia (CD11b^+^CD45^low^) (**Figures S4C–F**). Next, as shown in the scheme in **Figure 5A**, RBP-J^cKO^ and control mice were fed the PLX5622 diet or standard diet for 7 days and then treated with MPTP to induce the PD model and fed the PLX5622 diet or SD for another 7 consecutive days. Immunofluorescence staining showed that mice with continuous administration of the PLX5622 diet showed a remarkable decrease in the number of microglia and exhibited improved DA neurodegeneration in both RBP-J–deficient and control PD mice (**Figures 5B, C**). In addition, FACS results showed that CD11b^+^CD45^lo^ microglia and CD11b^+^CD45^hi^-infiltrated IMs, especially Ly6C^lo^CD11b^+^CD45^hi^-infiltrated IMs, decreased significantly in the PLX5622-treated groups (**Figures 5D–F**, **Figures S5**). As expected, MHC II^+^ activated microglia were decreased remarkably in PLX5622-treated RBP-J–deficient and control mice (**Figures 5G, H**). Correspondingly, the serum TNF-α and IL-6 but not TGF-β were decreased obviously in PLX5622-treated RBP-J–deficient and control mice (**Figure 5I**). Collectively, these results demonstrated that myeloid-specific RBP-J deficiency could ameliorate DA neurodegeneration by reducing MHCII^+^ microglial activation.

### RBP-J–deficient primary microglia exhibited reduced proinflammatory cytokine secretion through NF-κB signaling

**Figure 6:**
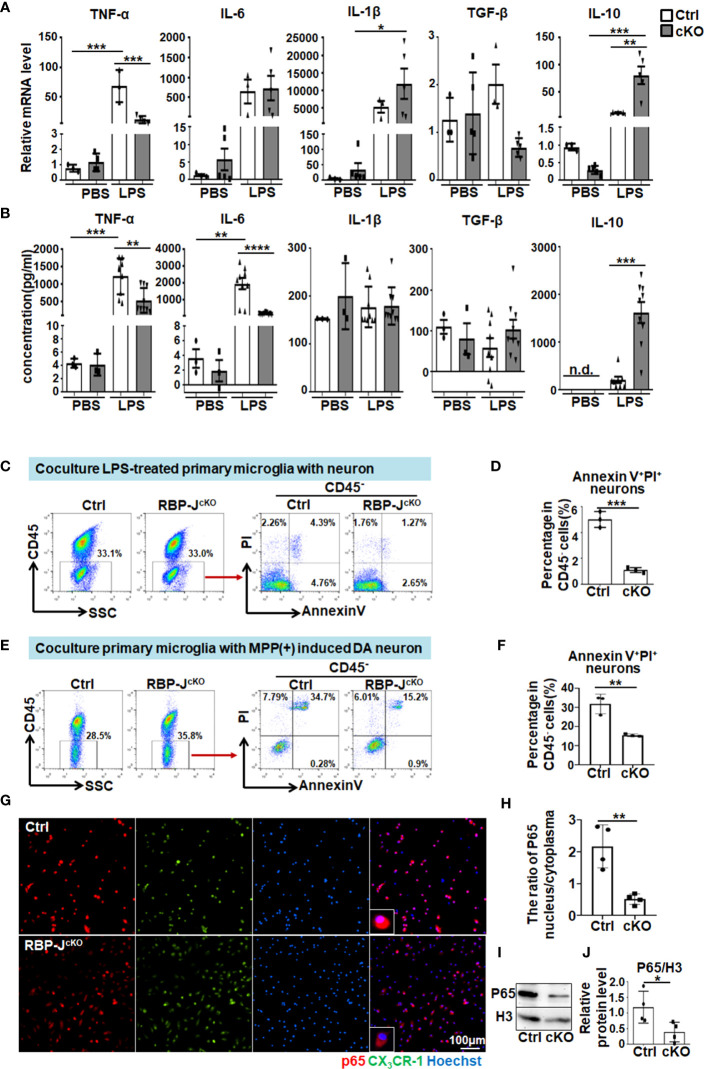
RBP-J–deficient primary microglia exhibited reduced proinflammatory cytokine secretion through NF-κB signaling. **(A)** Primary microglia were isolated from RBP-J^cKO^ and control mice and then cultured and stimulated with LPS or PBS (100 ng/mL) for 24 h. After that, cells were collected for RNA extraction, and the relative mRNA expression levels of TNF-α, IL-1β, IL-6, IL-10, and TGF-β were determined by RT-PCR [primary microglia from Ctrl mice: n = 3; primary microglia from cKO mice: TNF-α (n = 4), IL-6 (n = 5), IL-1β (n = 5), TGF-β (n = 5), and IL-10 (n = 5)]. **(B)** The protein levels of TNF-α, IL-1β, IL-6, IL-10, and TGF-β in cultured medium collected from primary microglia in **(A)** were detected by ELISA (n = 3 in groups treated with PBS, n = 9 in groups treated with LPS except IL-10, in which n = 8 in LPS-Ctrl group). **(C)** LPS-stimulated primary microglia from RBP-J^cKO^ or control mice were cocultured with HT-22 cells for 24 h, and, then, the apoptotic HT-22 cells in CD45-negative cells were examined by Annexin V/PI staining. **(D)** The AnnexinV^+^PI^+^ apoptotic HT-22 cells in **(C)** were quantitatively compared (n = 3). **(E)** Cocultured primary microglia from RBP-J^cKO^ or control mice with MPP+ (1 μM)–treated SH-SY5Y for 48h, and, then, the apoptotic SH-SY5Y cells in CD45-negative cells were examined by AnnexinV/PI staining. **(F)** The Annexin V^+^ PI^+^ apoptotic HT-22 cells in **(E)** were quantitatively compared (n = 3). **(G)** Primary microglia were isolated from RBP-J^cKO^/CX3CR1^GFP/+^ and Ctrl/CX3CR1^GFP/+^ mice and then cultured on coverslips overnight. After stimulation with LPS (100 ng/mL) for 6 h, cells on coverslips were subjected to immunofluorescence staining with anti-p65 antibody and Hoechst. **(H)** The ratio of P65 nucleus/cytoplasm was calculated depending on the fluorescence intensity (n = 4). **(I)** Primary microglia in **(A)** were lysed, and, then, the nucleic and cytoplasmic proteins were extracted. The expression of P-p65 in the nucleic was measured by Western blot, with H3 as an internal control (n = 4). **(J)** The relative protein level of nuclear p65 was quantitatively compared (n = 4). One-way ANOVA with Tukey’s multiple comparisons test was used for the statistical analyses. Bars = mean ± SD. n.d., not detectable. *P < 0.05; **P < 0.01; ***P < 0.001; ****P < 0.0001.

To further explore the mechanism of Notch signaling-regulated microglial activation in PD progression, primary microglia were isolated from RBP-J^cKO^ and control mice according to the described protocol (28, 51). The purity of isolated primary microglia was approximately 96.7%, as determined by immunofluorescence staining and FACS assay (**Figures S6A, B**). Then, the primary microglia were stimulated with LPS for 24 h, and the mRNA and protein levels of proinflammatory factors, including TNF-α, IL-6, and IL-1β, as well as anti-inflammatory cytokines, including TGF-β and IL-10, were measured by qRT-PCR, ELISA, and immunofluorescence staining. The results showed that the protein levels of TNF-α and IL-6 decreased markedly, whereas the IL-10 level increased significantly in RBP-J^cKO^ microglia (**Figures 6A, B**, **Figures S6C, D**). Furthermore, we first observed the effect of LPS-stimulated microglia on dopamine neurons by coculture experiments. The results showed that CD45^−^AnnexinV^+^PI^+^ apoptotic neurons decreased remarkably in RBP-J–deficient microglia, which might be due to fewer proinflammatory cytokines and more anti-inflammatory cytokines secreted by RBP-J^cKO^ microglia after LPS stimulation (**Figures 6C, D**). Because of the cytotoxin effect of LPS on primary microglia, we further performed the coculture experiments using primary microglia and MPP(+)-induced DA neurons as shown in **Figures 6E, F**. The results were consistent with the coculture experiment using LPS-treated microglia and neuron. Meanwhile, bone marrow–derived macrophages (BMDMs) were cultured as previously described (34) and stimulated with LPS for 24 h; then, the mRNA and protein levels of inflammatory factors were detected, and the results showed that RBP-J deficiency in BMDMs could not alleviate inflammatory response significantly (**Figures S7A, B**); in line with this, the experiment that cocultures BMDMs with DA neurons showed a little effect on the apoptosis of DA neurons (**Figures S7C, D**). These results were consistent with the previous reports that microglia and BMDM showed different gene profiles in neurodegeneration (52, 53). As Notch signaling can cooperate with Toll-like receptors (TLR) signaling to defend against pathogen infection through NF-κB signaling, we further examined the downstream molecules of NF-κB signaling using immunofluorescence staining and Western blotting with an anti-p65 antibody. All results showed that the nuclear expression of p65 was significantly reduced in RBP-J–deficient microglia after LPS stimulation (**Figures 6G-J**). Together, these results indicated that myeloid-specific blockade of Notch signaling could participate in PD progression by mainly affecting the microglia-mediated neuroinflammation through NF-κB signaling.

## Discussion

Microglia are the most abundant innate immune cells in the CNS that can mediate synaptic pruning and remodeling by interacting with neurons in physiological and pathological conditions (54–56). In the past few decades, the heterogeneity of microglial phenotype and function in neurodegenerative diseases such as PD has received great attention, but the mechanisms that regulate microglia from the physiological state to the pathological state are still unclear. Notch signaling has been reported to play a critical role in regulating microglial activation and neuroinflammation-related disorders such as cerebral ischemia and epilepsy (57, 58). Our previous studies have further shown that inhibition of Notch signaling in myeloid cells could significantly alleviate spinal cord injury or Guillain-Barré syndrome by reducing proinflammatory macrophage polarization or promoting inflammatory monocyte conversion (23, 24). In this study, we found that Notch signaling can be greatly activated in microglia of the MPTP-induced acute PD mouse model accompanied by decreased TH^+^ neurons in the SNpc. As expected, myeloid-specific blockade of Notch signaling inhibited DA neuron death and improved mouse motor behavior by reducing MHCII^+^ microglial activation and IM infiltration. Moreover, we demonstrated that myeloid-specific RBP-J deficiency could attenuate PD progression by reducing inflammatory factor secretion through NF-κB signaling. On the basis of our findings, targeting Notch signaling in myeloid cells might be a potential therapeutic strategy for neuroinflammation-related diseases, including PD, in the future.

The brain macrophage population demonstrates increasing heterogeneity and plasticity following the application of single cell RNA sequencing (scRNA-seq), which includes tissue-resident microglia, border-associated macrophages, and recruited monocyte-derived macrophages (14, 15, 59). Although the contribution of both activated microglia and infiltrated monocytes in neuroinflammation has been widely studied, the conclusion remains controversial (46–48, 60). Here, using myeloid-specific RBP-J–deficient mice combined with an acute PD model, we found that Notch signaling blockade in myeloid cells could ameliorate the symptoms of murine PD. On the one hand, Notch signaling blockade could regulate microglial activation as the total microglia number was not affected; on the other hand, Notch signaling blockade could reduce monocyte infiltration. In general, the CCR2–CCL2 axis is a popular chemokine axis for recruiting peripheral monocytes into the CNS during neuroinflammation (61, 62). Our recent studies have shown that myeloid-specific Notch signaling disruption could alleviate renal or lung fibrosis progression by regulating monocyte-derived macrophage recruitment *via* the CCR2–CCL2 axis (37, 38). Unexpectedly, in the current study, CCR2 knockout did not result in significantly decreased IMs in RBP-J^cKO^ PD mice and contributed less to the increased numbers of TH^+^ DA neurons in RBP-J^cKO^ PD mice, suggesting that Notch signaling blockade in myeloid cells alleviated DA neurodegeneration independent of CCR2^+^ monocyte recruitment. However, Ly6C^lo^ IMs decreased significantly in RBP-J^cKO^ PD mice, which was reminiscent of the contribution of Ly6c^lo^CX3CR1^hi^CCR2^lo^ patrolling monocytes to infiltrated macrophages in MPTP-treated mice (48, 63). Whether Notch signaling in myeloid cells could regulate these patrolling monocytes involved in PD progression still needs to be investigated.

Microglial activation has been demonstrated to be a key regulator of PD pathogenesis (10). MHC II is a hallmark of microglial activation and was first reported by McGeer et al. in 1988. They found large numbers of Human leukocyte antigen DR (HLA-DR)–positive reactive microglia (macrophages) along with Lewy bodies in the substantia nigra of patients with PD (64). Recently, Williams et al. further suggested that targeting MHC II expression by shRNA against *CIITA* in microglia could attenuate inflammation and neurodegeneration in an α-synuclein model of PD (65). In our study, we also found that, regardless of whether CCR2 was knocked out, the total number of MHC II^+^ activated microglia decreased significantly in RBP-J^cKO^ PD mice. Moreover, RBP-J–deficient microglia exhibited less proinflammatory factor secretion and neuronal apoptosis, suggesting that Notch signaling might dominantly regulate microglial activation involved in PD pathogenesis rather than CCR2-depenedent monocyte recruitment.

To address the role of microglia under pathological conditions, researchers usually adopt microglia depletion experiments using clodronate liposomes, anti-colony stimulating factor 1 receptor (CSF1R) antibodies, or CSF1R inhibitors. Intracerebral administration of clodronate liposomes into the brain parenchyma can cause macrophage apoptosis but can also damage other brain cells, including blood vessels (66). CSF1R is expressed on microglia/macrophages and is responsible for their survival and proliferation (67, 68). Although genetic deletion of CSF1R can be used to deplete microglia, other cells expressing CSF1R are often affected (69). Recently, one CSF1R inhibitor, PLX5622, has been largely assumed to be microglia-specific with few off-target effects, which have beneficial effects on motor and non-motor symptoms in a PD model (70). On the basis of 90% microglia depletion efficiency, we utilized a PLX5622-formulated diet to evaluate the contribution of RBP-J–deficient microglia to PD progression and found that microglia depletion could inhibit DA neurodegeneration. However, in addition to microglia depletion, in our system, we also found that CD11b^+^Ly6C^lo^ monocytes were depleted (data not shown). This phenomenon is consistent with a previous report in which CX3CR1^hi^ly6C^lo^ monocytes could be severely depleted by CSF1R inhibition, whereas CX3CR1^lo^ly6C^hi^ monocytes could not be depleted in the peripheral immune system (69). Because myeloid-specific RBP-J deficiency reduced the infiltration of Ly6c^lo^CXCR1^hi^ IMs in MPTP-treated mice (**Figures 3D, F**), the effect of PLX5622 on this population may not be an ideal method for investigating microglial function under a Notch signaling disruption background. In addition, the study of Orthgiess et al. founded that, targeting microglia *in vivo* using the LysM promoter is less efficient than that using the CX3CR1 promoter and neurons that exhibit LysM promoter activity (71). Therefore, in the future, more genetically modified mice, such as microglia-specific Notch-activated mice (CX3CR1-Cre or TMEM119-Cre combined with NICD ^stop-flox^), should be used to clarify the importance of Notch signaling in regulating microglial activation in PD progression.

In summary, our present study first demonstrates that myeloid-specific Notch signaling blockade can alleviate DA neurodegeneration in PD mice. The underlying cellular mechanism may be attributed to reduced MHC II^+^ activated microglia and infiltrated Ly6c^lo^CX3CR1^hi^ macrophages. Meanwhile, the molecular mechanism may be related to the alleviation of neuroinflammation regulated by NF-κB signaling.

## Data availability statement

The original contributions presented in the study are included in the article/**Supplementary Material**. Further inquiries can be directed to the corresponding author.

## Author contributions

S-QL, P-HL, and Y-YH performed experiments and analyzed the results. J-LZ, F-ZS, and FK took part in various aspects of the study and revised first draft. K-XR, T-XW, and LF participated in all animal experiments. FF performed data statistics and analysis. HH discussed the data. H-YQ designed the project, analyzed the data, and supported the study. S-QL and H-YQ wrote the manuscript. All authors contributed to the article and approved the submitted version.
