# Supplementary material for: Myeloid-specific blockade of notch signaling alleviates dopaminergic neurodegeneration in Parkinson’s disease by dominantly regulating resident microglia activation through NF-κB signaling
Source: Front Immunol. 2023 Aug 23;14:1193081. doi: 10.3389/fimmu.2023.1193081 (PMC10481959; doi:10.3389/fimmu.2023.1193081)
Supplement: Supplementary file 1 [file DataSheet_1.pdf]

Supplemental Figures

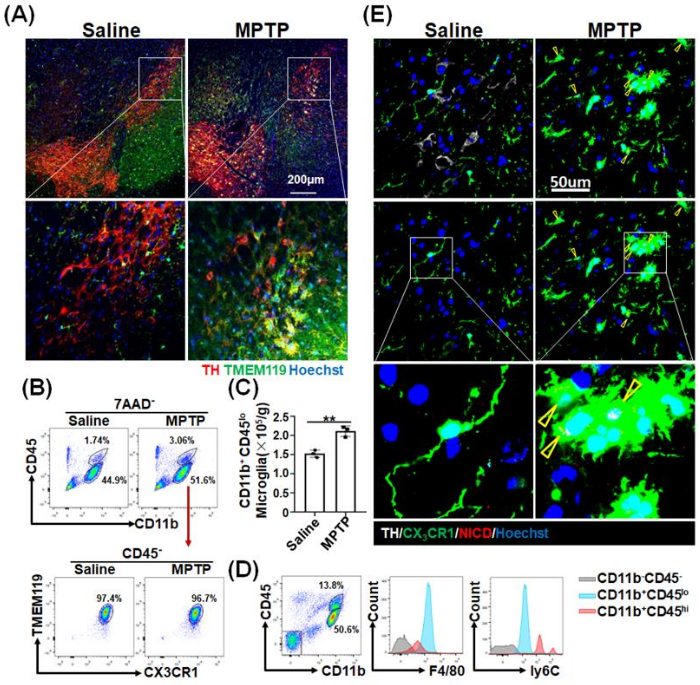

Figure S1. Notch signaling was activated in MPTP-treated PD model using CX3CR1$^{GFP/+}$ mice. (A) Representative immunofluorescence images of TH and TMEM119 staining in the substantia nigra pars compacta (SNpc) of C57BL/6 mice suffering from PD and control mice. (B) Single-cell suspensions were prepared from the brains of PD and control (saline) mice. Microglia were analyzed by FACS with different surface markers including CD45, CD11b, CX3CR1and TMEM119. (C) The number of CD11b^{+}CD45^{lo} microglia in (B) were analyzed and quantitively compared (n=3). (D) To distinguish CD11b^{+}CD45^{lo} microglia and CD11b^{+}CD45^{hi} IMs with F4/80 and Ly6C staining by FACS. (E) Representative immunofluorescence images of TH and NICD staining in SN of CX3CR1$^{GFP/+}$ mice after treatment with MPTP and saline respectively. The images were acquired under a confocal fluorescence microscope. Student's t test was used for the statistical analyses. Bars=mean ± SD. **, P < 0.01.

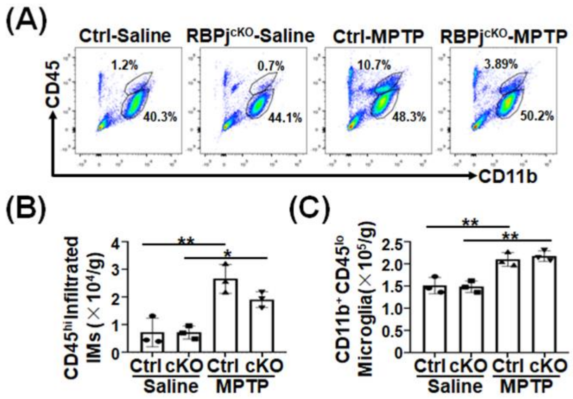

Figure S2. MPTP treatment induced microglia proliferation and IMs infiltration in both RBP-J^{cKO} and control mice. (A) Single cell suspensions were prepared and analyzed by FACS from the brain of RBP-J^{cKO} and control mice after MPTP or saline treatment. (B-C) The number of IMs (B) and microglia (C) in (A) were quantitively compared among each group (n=3). One-way ANOVA with Tukey's multiple comparison test were used for the statistical analyses. Bars=mean ± SD. *, $P < 0.05$; **, $p< 0.01$.

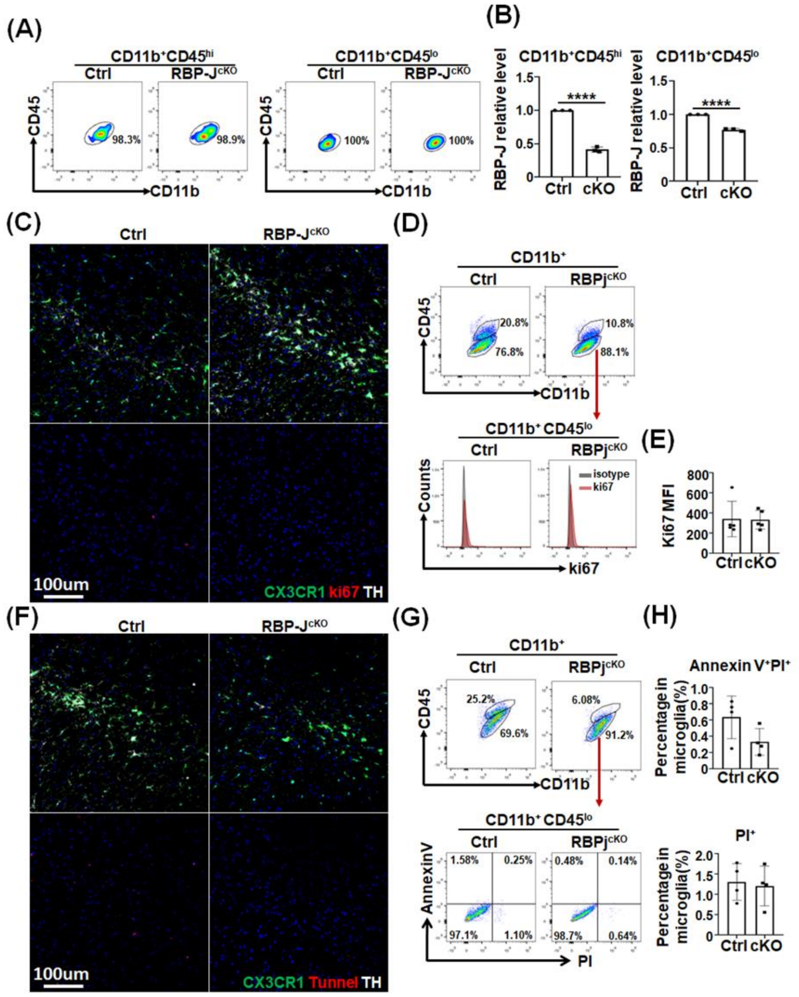

Figure S3. Myeloid-specific RBP-J deficiency had little effects on proliferation and apoptosis of microglia of PD mouse. (A) CD11b^{+}CD45^{lo} microglia were sorted and the purity was analyzed by FACS. (B) The knockout efficiency of RBP-J in harvested CD11b^{+}CD45^{hi} IMs and resident CD11b^{+}CD45^{lo} microglia in (A) was confirmed by q-PCR. (C) Representative immunofluorescence images of Ki67 staining in the SN of RBP-J^{cKO} and control (Ctrl) mice after MPTP treatment. (D) Single cell suspensions were prepared from the brain and Ki67^{+} cells were analyzed by FACS. (E) Mean fluorescence intensity (MFI) of Ki67 in microglia were quantitatively compared (n=5). Student's t test was used for the statistical analyses. Bars=mean ± SD. ****$p< 0.0001$.

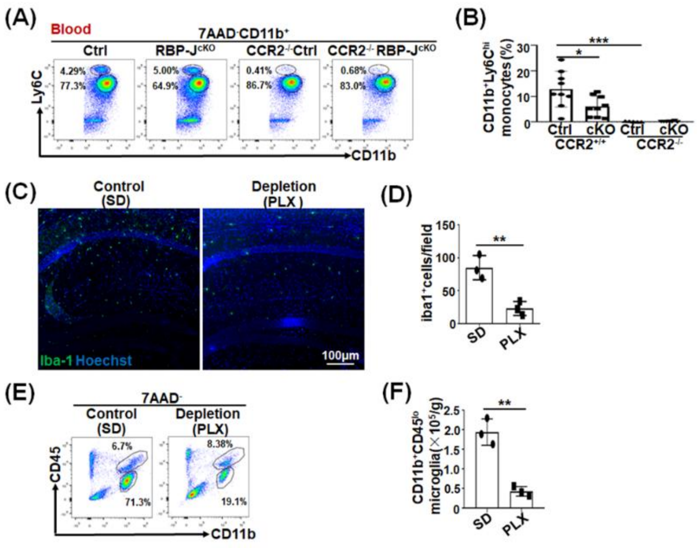

Figure S4. The efficiency of CCR2^{+} monocytes and microglia depletion were confirmed, respectively. (A) Ctrl, RBP-J^{cKO}, CCR2$^{-/-}$ and RBP-J^{cKO}/CCR2$^{-/-}$ mice were treated intraperitoneally with MPTP·HCl to induce the acute PD model. Single cell suspensions were prepared from the blood and Ly6c labeled monocytes were analyzed by FACS; (B) The percentage of CD11b^{+}ly6C^{hi} monocytes in (A) were quantitatively compared (n=8 in Ctrl and RBP-J^{cKO}, n=5 in RBP-J^{Ctrl}/CCR2$^{-/-}$, n=7 in RBP-J^{cKO}/CCR2$^{-/-}$); (C) The Iba-1^{+} microglia was detected using immunofluorescence staining on brain sections from standard diet-fed and PLX5622 diet-fed, respectively; (D) The Iba-1^{+} microglia was counted in the fields in (C) and compared in order to judge the depletion efficiency of microglia in mice fed with PLX5622 diet (n=3); (E) Flow cytometry analysis of microglia depletion efficiency; (F) The number of CD11b^{+}CD45^{lo} microglia in (E) were quantitively compared (n=3). One-way ANOVA with Tukey's multiple comparison test or Student's t test were used for the statistical analyses. Bars=mean ± SD. *, $P < 0.05$; **, $p < 0.01$; ***$p < 0.001$.

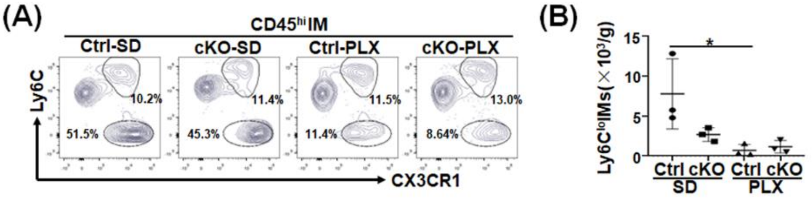

Figure S5. Ly6C^{-}CX3CR1^{+}IMs were dramatically reduced after PLX5622 treatment. (A) Single cell suspensions were prepared from the brain of RBP-J^{cKO} and control PD mice fed with different diet, respectively. Lyc^{hi} or Ly6c^{lo} CD11b^{+}CD45^{hi} infiltrated inflammatory macrophages were analyzed; (B) The total cell number of Ly6C^{-}CX3CR1^{+}IMs in brain were calculated and quantitively compared(n=3). One-way ANOVA with Tukey's multiple comparison test was used for the statistical analyses. Bars=mean ± SD. *, $P < 0.05$.

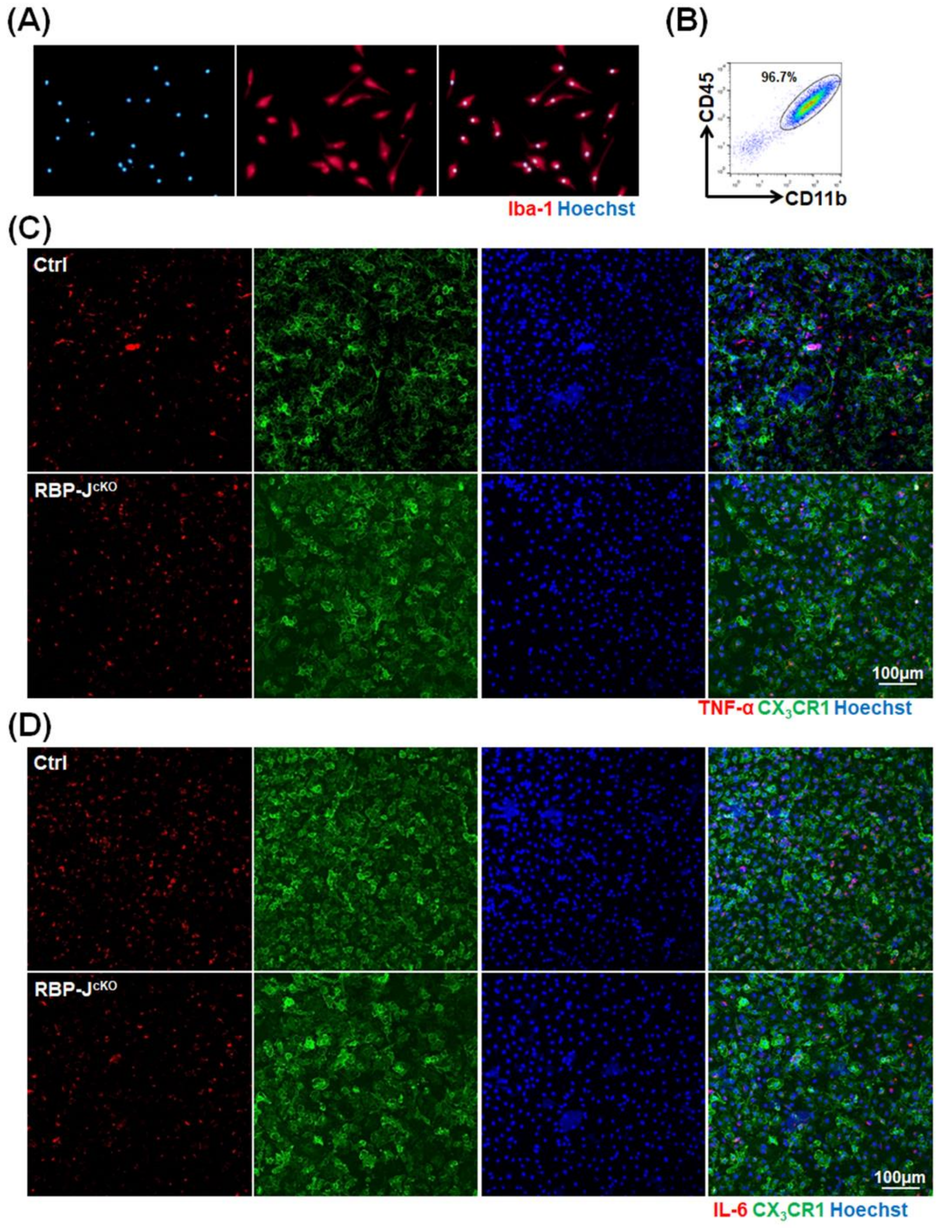

Figure S6. Primary microglia isolated from myeloid-specific RBP-J deficient mice showed reduced TNF-a and IL-6 expression. (A) To evaluate the primary microglia isolated from C57BL/6 mice with anti-Iba-1 antibody using immunofluorescence staining; (B) To evaluate the primary microglia isolated from C57BL/6 mice by FACS with CD11b and CD45 staining; (C-D) Representative immunofluorescence images of TNF-α and IL-6 staining in primary microglia isolated from RBP-J^{cKO} and control mice after LPS treatment for 24h.

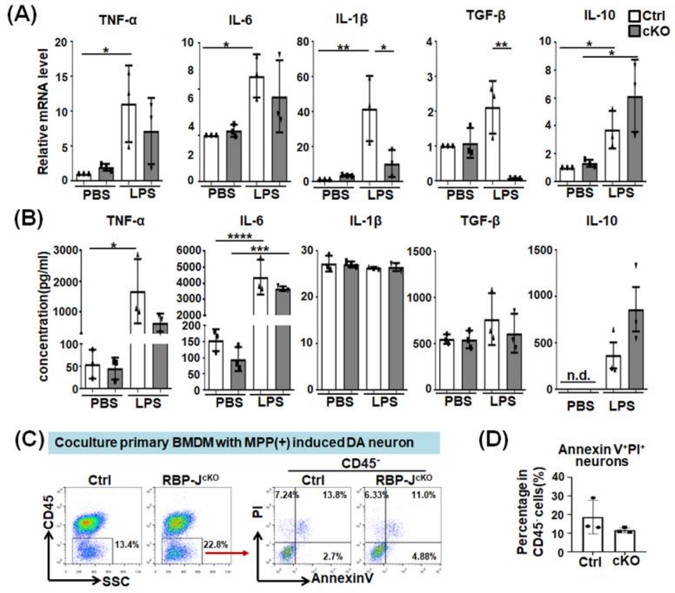

Figure S7. RBP-J-deficient BMDM showed little effect on proinflammatory cytokine secretion and apoptosis of DA neurons. (A) BMDM were isolated from RBP-J^{cKO} and control mice respectively, and then cultured and stimulated with 100ng/ml LPS or PBS for 24h. After that, Cells were collected for RNA extraction and the relative mRNA expression of TNF-α, IL-1β, IL-6, IL-10 and TGF-β were determined by RT-PCR(n=3); (B) The protein level of TNF-α, IL-1β, IL-6, IL-10 and TGF-β in cultured medium collected from primary microglia in (A) were detected by ELISA (n=3); (C) Co-cultured BMDM from RBP-J cKO or control mice with MPP+(1μM) treated SH-SY5Y for 48h, and then the apoptotic SH-SY5Y cells in CD45 negative cells were examined by AnnexinV/PI staining; (D) The Annexin V^+ PI^+ apoptotic SH-SY5Y cells in (C) were quantitively compared (n=3); One-way ANOVA with Tukey's multiple comparison test were used for the statistical analyses. Bars=mean ± SD. BMDM, bone marrow-derived macrophages. n.d., not detectable. *, $P < 0.05$; **, $p < 0.01$; ***, $p < 0.001$; ****$p < 0.0001$.
