# Supplementary material for: Myeloid-specific blockade of notch signaling alleviates dopaminergic neurodegeneration in Parkinson’s disease by dominantly regulating resident microglia activation through NF-κB signaling
Source: Front Immunol. 2023 Aug 23;14:1193081. doi: 10.3389/fimmu.2023.1193081 (PMC10481959; doi:10.3389/fimmu.2023.1193081)
Supplement: Supplementary file 2 [file DataSheet_2.pdf]

Supplementary Material

TABLE S1. Primer sequence for mice genotyping and DNA amplification

Name	Purpose	Sequence
RBP-J-F	PCR	5'-GTTCTTAACCTGTTGGTCGGAACC
RBP-J-WT-R	PCR	5'-GCTTGAGGCTTGATGTTCTGTATTGC
RBP-J-floxed-R	PCR	5'-ACCGGTGGATGTGGAATGTGT
Cre-mutant	PCR	5'-CCCAGAAATGCCAGATTA
Cre-common	PCR	5'-CTTGGGCTGCCAGAATTTCTC
Cre-Wild type	PCR	5'-TTACAGTCGGCCAGGCTGAC
CX3CR1-mutant	PCR	5'-CTCCCCCTGAACCTGAAAC
CX3CR1-Wild type	PCR	5'-GTCTTCACGTTCGGTCTGGT
CX3CR1-common	PCR	5'-CCCAGACACTCGTTGTCCTT
CCR2-mutant	PCR	5'-CCTTCTATCGCCTTCTTGACG
CCR2-Wild type	PCR	5’-CACAGCATGAACAATAGCCAAG
CCR2-common	PCR	5’-CCACAGAATCAAAGGAAATGG
RBP-J exon (6-7) F	q-PCR	AAGGATAACCATTGGTCTGTTTCCACC
RBP-J exon (6-7) R	q-PCR	GACAGTCTGCCCGTAATGGATGTAGC
RBP-J exon (8-9) F	q-PCR	ATGACCAGTGAATGGCCTCTGTCC
RBP-J exon (8-9) R	q-PCR	CATATGAACCCAGCTTCCTGCATGT

*F, forward; R, reverse.

Table S2. Antibodies and related reagents used in this study.

Name	Supplier	Catalog #	Titration
FITC CD11b	ebioscience	11-0112	1/100
BV510 CD45	Biolegend	103137	1/200
PE CX3CR1	Biolegend	149006	1/800
PE-CY7 MHC II	Biolegend	107629	1/200
APC Ly6C	Biolegend	128015	1/400
Biotin Ly6G	Biolegend	127603	1/1000
BV421 CCR2	Biolegend	150605	1/400
APC-CY7 streptavidin	Biolegend	405208	1/800
PE-CY7 TMEM119	ebioscience	25-6119-82	1/800
Biotin MHC II	BD	553607	1/1600
BV421 streptavidin	Biolegend	405226	1/1000
PE-ki67	Biolegend	652403	1/400
Rab anti-iba1	wako	234003	1/1000
Rab anti-TMEM119	abcam	ab209064	1/200
Rab anti-p65	abcom	ab32536	1/1000
Mouse anti-TH	Sigma	T1299	1/10000
Rab anti-NICD	abcam	Ab8925	1/200
Mouse anti-H3	CST	3638	1/1000
Cy3 goat anti-rabbit IgG	Boster Bio Tec	BA1032	1/500
Cy3 goat anti-mouse IgG	Life technology	A10521	1/1000
AF 488 anti-rabbit IgG	Life technology	35552	1/1000
Cy5 goat anti-mouse IgG	Life technology	A10524	1/1000
